# Reduction of secondary bile acids following microbiome pre-conditioning was associated with reduced breast cancer progression

**DOI:** 10.1038/s41598-026-59669-3

**Published:** 2026-07-31

**Authors:** Margaret S. Bohm, Johnathan R. Yarbro, Clàudia Font Torres, Zeid T. Mustafa, Mehdi Chaib, Tahliyah S. Mims, Sydney C. Joseph, Joel H. Elasy, Laura M. Sipe, Katherine L. Cook, Joseph F. Pierre, Liza Makowski

**Affiliations:** 1 Department of Microbiology, Immunology, and Biochemistry, College of Medicine, The University of Tennessee Health Science Center, Memphis, TN 38163 USA; 2 Department of Medicine, Division of Hematology and Oncology, College of Medicine, The University of Tennessee Health Science Center, Memphis, TN 38163 USA; 3 Department of Pharmaceutical Sciences, College of Pharmacy, The University of Tennessee Health Science Center, Memphis, TN 38163 USA; 4 Department of Nutritional Sciences, College of Agricultural and Life Sciences, University of Wisconsin-Madison, Madison, WI 53706 USA; 5 Department of Biology, University of Mary Washington, Fredericksburg, VA 22401 USA; 6 Department of Cancer Biology, Wake Forest University School of Medicine, Winston-Salem, NC 27157 USA; 7 UTHSC Center for Cancer Research, College of Medicine, The University of Tennessee Health Science Center, Memphis, TN 38163 USA; 8 19 South Manassas Cancer Research Building Room 322, Memphis, TN 38163 USA

**Keywords:** Cancer, Microbiology, Oncology

## Abstract

Breast cancer is the most common cause of cancer-related death in women worldwide. We previously reported that a primary bile acid mimetic reduced tumor progression in vivo. Here, we hypothesized that elevating primary bile acids through preconditioning of the gut microbiome may be an unexplored opportunity to improve breast cancer. We utilized the antibiotic vancomycin previously reported to deplete Gram-positive, anaerobic microbes that convert primary bile acids to secondary bile acids. Preconditioning with vancomycin significantly delayed tumor onset, decreased burden, promoted regression, and reduced the number of mice with lung colonization. Vancomycin reduced overall microbiome diversity and specific microbes in *Clostridium Cluster XIVa*, which associated with increased primary bile acid levels. We report that vancomycin significantly associated with elevated circulating bile acid tauro-alpha/beta-muricholic acid and reduced secondary bile acids that correlated with worsened tumor outcomes. Flow analysis of tumor content and systemic immune responses was associated with immune changes consistent with anti-tumor responses, including conventional dendritic cells and activated CD8 + T cells. In sum, we demonstrated that manipulation of the bile acid pools associated with improved breast cancer outcomes. The altered bile acids corresponded to an elevation of key immune cells, which may contribute to protection from primary breast cancer progression and lung colonization.

**Supplementary Information:**

The online version contains supplementary material available at https://doi.org/10.1038/s41598-026-59669-3.

## Introduction

Breast cancer (BC) is the leading cause of cancer incidence and mortality in women worldwide^1^. The gut microbiome plays a critical role in the development, progression, and response to treatment for many types of cancer^2,3^, including breast^4,5^. One potential mechanism that may be critical to microbial impacts on tumor outcomes is changes to the gut microbiome composition and associated shifts in host-microbiome interactions and microbially derived metabolites (MDMs) associated with microbiome dysbiosis^6–9^. MDMs include secondary bile acids, short chain fatty acids, amino acids including branched chain amino acids, tryptophan metabolites, and other small molecules^10^. These metabolites are produced or modified by commensal gut microbes to act locally within the gut and circulate systemically. MDMs affect microbial and host energy metabolism, gut mucosal integrity, gut-associated immune maturation, and systemic immune homeostasis^11–^^13^.

The role of bile acid signaling in cancer is complex, not well defined, and can be either tumor-promoting or tumor-suppressive in multiple cancer types^14^. Bile acids are known modulators of both the innate and adaptive immune system, supporting either anti-tumor immunity or immunosuppression depending upon the bile acid profile and tissue-specific disease context^14–18^. Ma et *al*. have demonstrated that secondary bile acids are deleterious and exacerbate tumor outcomes in hepatocellular carcinoma^18^. Evidence of elevated bile acid metabolism is associated with biomarkers of reduced BC growth and proliferation as well as increased patient survival across several cohorts^19^. Bile acids have been identified in the breast and breast tumors which associate with patient survival^20^. Furthermore, we have shown that a bile acid mimetic reduced BC progression in vivo and migration in vitro^21^. However, little is known about the impact of specific circulating bile acids on BC progression.

The gut microbiome plays a critical role in regulating the composition of the bile acid pool. Modulation from primary to secondary bile acids and conjugation or deconjugation to generate specific bile acids is conducted by commensal gut microbes^22,23^. Patients with higher expression of the nuclear hormone receptor Farnesoid X Receptor (FXR, (*Nr1h4*) in breast tumors display favorable survival outcomes in aggressive BC subtypes^21^. Therefore, we tested if manipulating the gut microbiome to elevate primary bile acids could impact key cells typically associated with anti-tumor immunity to reduce BC burden. We report that using a Gram-positive targeting antibiotic, vancomycin^24^, to ablate microbes including those in the *Clostridium Cluster XIVa* associated with elevated primary bile acid tauro-α/β-muricholic acid as well as decreased secondary bile acids deoxycholic acid and taurohyodeoxycholic acid. Vancomycin treatment was associated with delayed tumor onset, slowed BC progression, elevated BC regression, and reduced lung colonization in an experimental lung colonization model potentially via activation of key immune cells through the classical dendritic cell (cDC)-CD8+ T cell cross-presentation axis.

## Results

### Gut microbiome pre-conditioning with vancomycin reduced BC tumor burden without impacting body weight

**Fig. 1:**
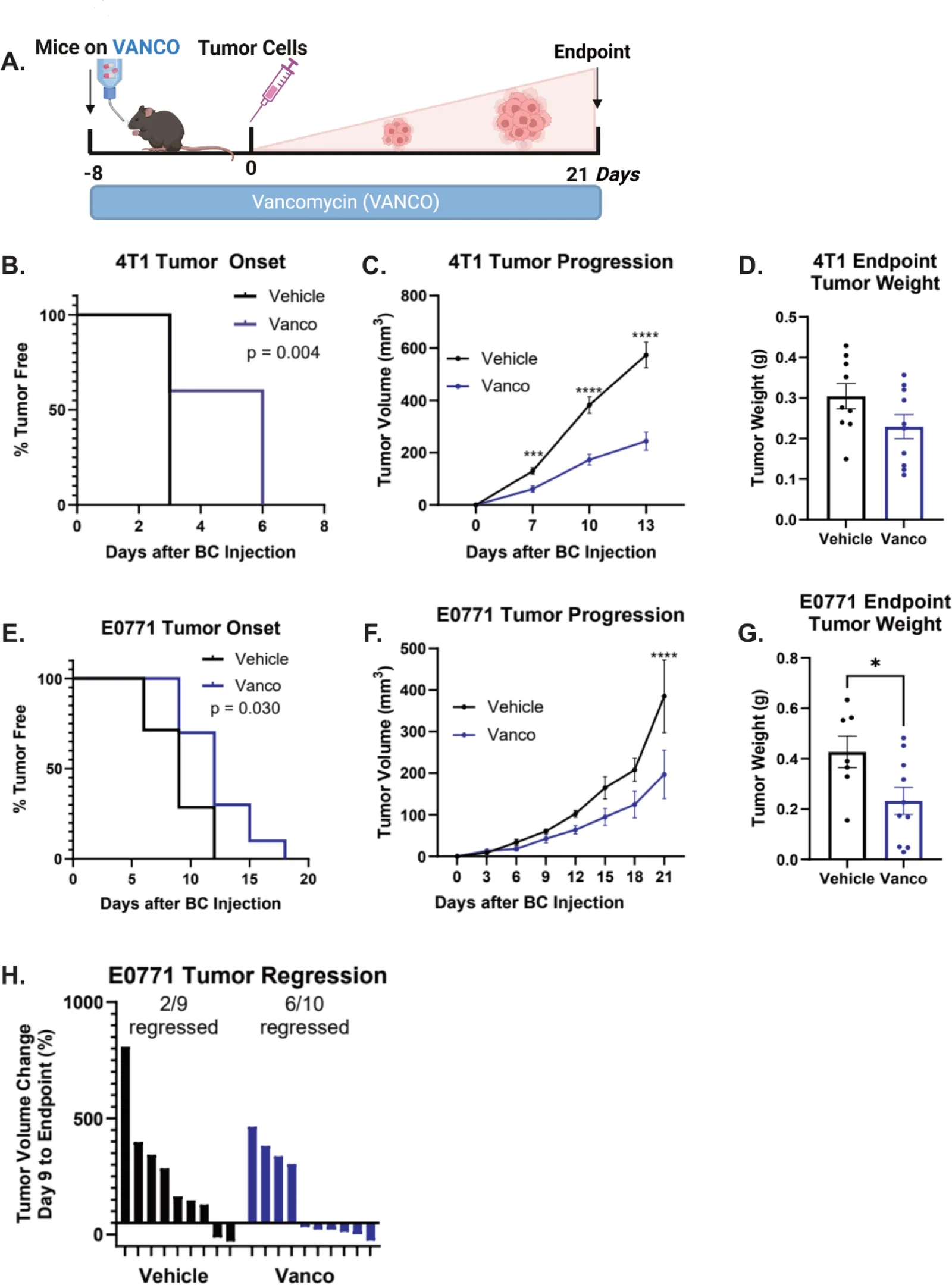
Vancomycin reduced breast cancer tumor burden.(**A**) Study Design: Female BALB/cJ or C57BL/6J mice were placed on water containing vancomycin (1 g/L) or plain sterile water for 8 days. 4T1 and E0771 breast cancer cells, respectively, were injected into the 4^th^ left mammary fat pad on day 0, and tumor progression was monitored until endpoint. (**B**) 4T1 tumor onset is reported as day tumors were measurable by digital caliper and presented as a Kaplan Meier survival plot with significance determined by logrank test. N = 10 per group. (**C**) 4T1 tumor progression was measured by digital caliper every 3 days until endpoint and is presented as mean ± SEM with multi-effects analysis and Fisher’s LSD correction. (**D**) Mass of excised 4T1 tumors was measured at study endpoint and is presented as mean ± SEM with Student’s *t*-test. (**E**) E0771 tumor onset is reported as day tumors were measurable by digital caliper and presented as a Kaplan Meier survival plot with significance determined by logrank test. N = 7-10 per group. (**F**) E0771 tumor progression was measured as in 4T1 model above. (**G**) Mass of excised E0771 tumors was measured at study endpoint and is presented as mean ± SEM with Student’s *t*-test. (**H**) E0771 tumor regression presented as percent change in tumor volume from day 9 to endpoint is shown as rank-sorted treatment groups. Tumors are considered to be regressing if the percent change in volume is less than 50%. The number of mice with regressed tumors is shown out of the total. *p < 0.05,***p < 0.001, ****p < 0.0001. Study cartoon was created with BioRender.

To investigate the role of bile acids and bile acid pool compositions in BC progression, female BALB/cJ and C57BL/6J mice were purchased from The Jackson Laboratory and treated with vancomycin or vehicle control. Vancomycin was introduced via drinking water as shown in the study schema (Fig. 1 A). BC cell injections, 4T1 or E0771 cells into BALB/cJ or C57BL/6J mice, respectively, occurred following one week of vancomycin treatment. Mice remained on vancomycin or vehicle (water) for the duration of the study period. No changes were observed between groups in body weight over time (Supplemental Figs. 3 A, 3D), with no differences in various adipose tissue depots, spleen, or liver weights observed at endpoint in C57BL/6J mice (Supplemental Figs. 3E).

Vancomycin pre-conditioning significantly delayed tumor onset in the aggressive 4T1 model, with palpable tumors present in all vancomycin-treated mice by day 6 post injection compared with day 3 in vehicle-treated mice (Fig. 1B). Delayed tumor onset was also observed in the E0771 BC model (Fig. 1E). Once tumors developed, vancomycin preconditioning associated with significantly slowed tumor progression in comparison to controls for both 4T1 and E0771 models (Fig. 1C and F). Blunted tumor progression resulted in reduced tumor weight at endpoint in both BC models (Fig. 1D and G, Supplemental Figs. 3B-C), though ulceration and necrosis in the 4T1 model resulted in less pronounced reductions in tumor weight as opposed to endpoint tumor volume. Vancomycin preconditioning also elevated tumor regression in the E0771 model (Fig. 1H). In the control group, 22% of the tumors regressed (2/9) while 60% of tumors (6/10) regressed in the vancomycin group (Fig. 1H). The groups graphed separately highlight the onset and progression of individual 4T1 (Supplemental Figs. 3B-C) and E0771 tumors (Supplemental Figs. 3G-H) throughout the course of the experiment. Taken together, mice receiving vancomycin preconditioning of the gut microbiome had significantly delayed tumor onset, reduced tumor progression and improved tumor regression compared to control groups across two BC models. Because vancomycin is a gut impermeable antibiotic that selectively targets Gram-positive bacteria, we next investigated microbial changes following treatment^24^.

*Vancomycin preconditioning reduced diversity in the commensal gut microbiota and reduced Clostridium Cluster XIVa.*

**Fig. 2:**
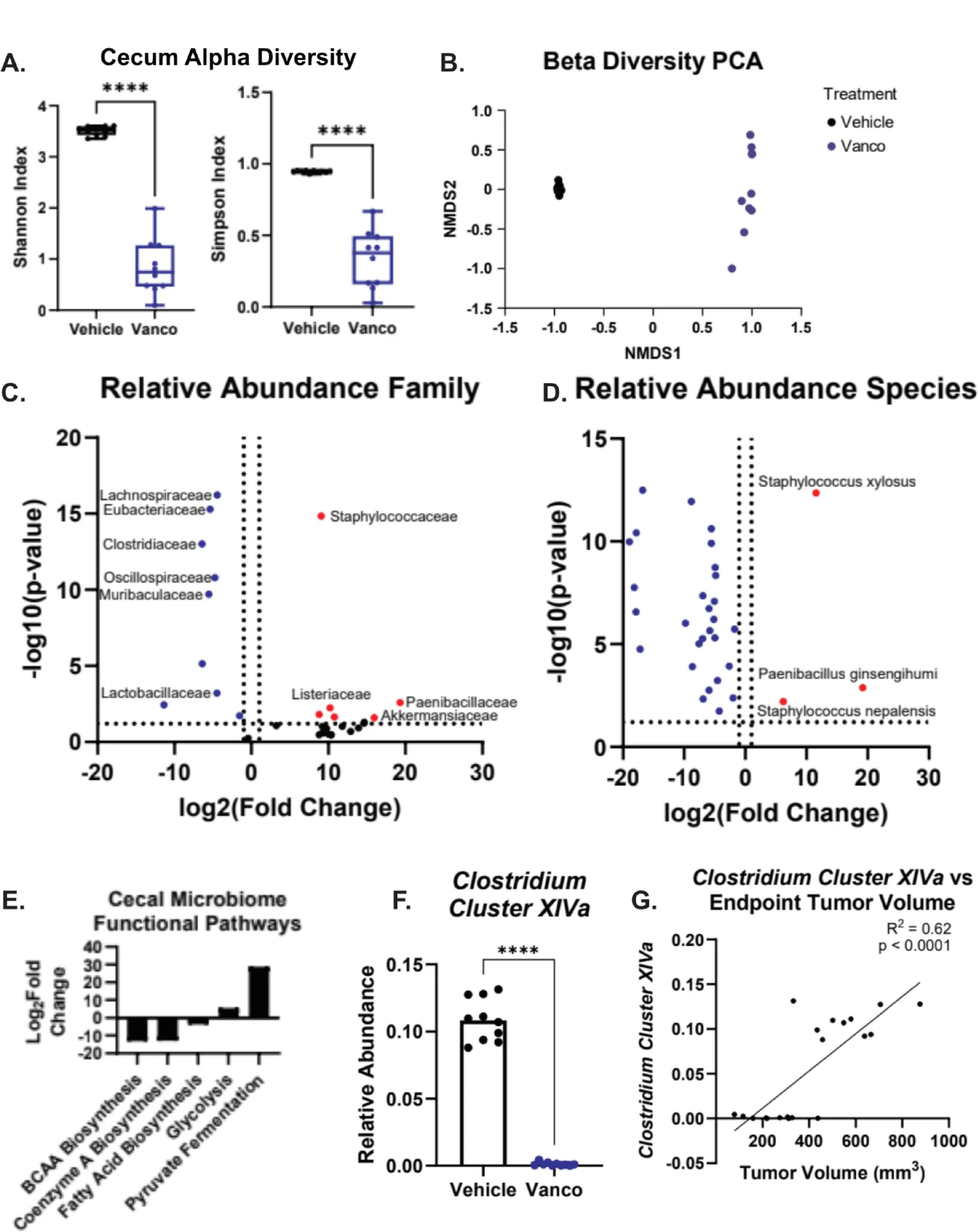
Vancomycin reduced diversity in the gut microbiota.(**A**) Microbial alpha diversity from metagenomic sequencing of cecal contents collected from 4T1 model at endpoint was quantified by Shannon and Simpson Indices. Data are presented as box and whisker plots with the boxes indicating the 25^th^ to 75^th^ percentiles, the whiskers extending to the maximum and minimum values, and the mean denoted by the line bisecting the box for N = 10 per group. (**B**) Beta diversity is shown by principal component analysis (PCA) of cecal contents with clustering determined by Bray-Curtis distances. N= 10 per group. (**C-D**) Volcano plots of significant differentially abundant microbial families (**C**) and species (**D**) in cecal contents at study endpoint plotted as vancomycin vs. vehicle groups with log_2_ fold change of greater than 1.5 and adjusted p value of less than 0.05 indicated by dot color, with red significantly elevated and blue significantly downregulated. (**E**) Selected functional pathways significantly impacted by vancomycin treatment were identified by the MetaCyc database using the Humann3 pipeline. (**F**) Relative abundance of *Clostridium Cluster XIVa* in cecal contents is reported. Data presented as mean ± SEM with Student’s *t-*test for N = 10 per group. (**G**) Relative abundance of *Clostridium Cluster XIVa* positively correlated with endpoint tumor volume by MaAsLin2 analysis, R^2^= 0.62, p < 0.0001, N = 20. ****p < 0.0001.

To determine the impact of vancomycin preconditioning on the commensal gut microbiome, we performed metagenomic whole genome shotgun sequencing on cecal contents collected from 4T1 tumor-bearing mice at study endpoint. Vancomycin preconditioning resulted in significantly reduced microbial alpha diversity by both Shannon and Simpson indices (Fig. 2A). Beta diversity scores were calculated with Bray-Curtis distances, and principal component analysis (PCA) clustering demonstrated significant differences in cecal content microbiome composition at study endpoint (Fig. 2B). The E0771 model at endpoint also indicated significant modulation of the microbiota following vancomycin preconditioning via beta diversity analysis with 16 S sequencing data (Supplemental Fig. 4A).

Comparison of the vehicle and vancomycin treated groups at the family level revealed significant modulation of many microbial families in the 4T1 model (Fig. 2C, Supplemental Fig. 5A) as well as in the E0771 model (Supplemental Fig. 4B). No cage effect was observed in any of the treatment groups. Among the decreased families are *Lachnospiraceae*, *Eubacteriaceae*, *Oscillospiraceae*, and *Lactobacillaceae* (Fig. 2C). Vancomycin treatment resulted in elevated abundance of taxa that may be beneficial for cancer outcomes as well, including families *Akkermansiaceae* and *Paenibacillaceae* (Fig. 2C)^25–28^. At the species level, there are elevations in two *Staphylococcucus* species and *Paenibacillus ginsengihumi* after vancomycin treatment (Figs. 2C-D). To determine potential impacts of microbial changes caused by vancomycin, functional analysis was conducted using the Humann3 pipeline and microbial pathways were defined by the MetaCyc database^29^. Vancomycin preconditioning significantly downregulated metabolic pathways, including pyruvate fermentation, amino acid biosynthesis, and glycolysis, which could impact the host immune response (a subset are shown in Fig. 2E, with all significant pathways shown in Supplemental Fig. 5B).

As expected^18^, vancomycin resulted in near complete ablation of members of *Clostridium Cluster XIVa* across both 4T1 and E0771 models (Fig. 2F, Supplemental Fig. 4C). Importantly, *Clostridium Cluster XIVa* associated significantly with increased tumor volume at endpoint in both models by microbiome multivariable associations with linear models (MaAsLin) analysis (Fig. 2G, Supplemental Fig. 4D).

### Vancomycin preconditioning associated with reduced bile acids associated with tumor burden

**Fig. 3:**
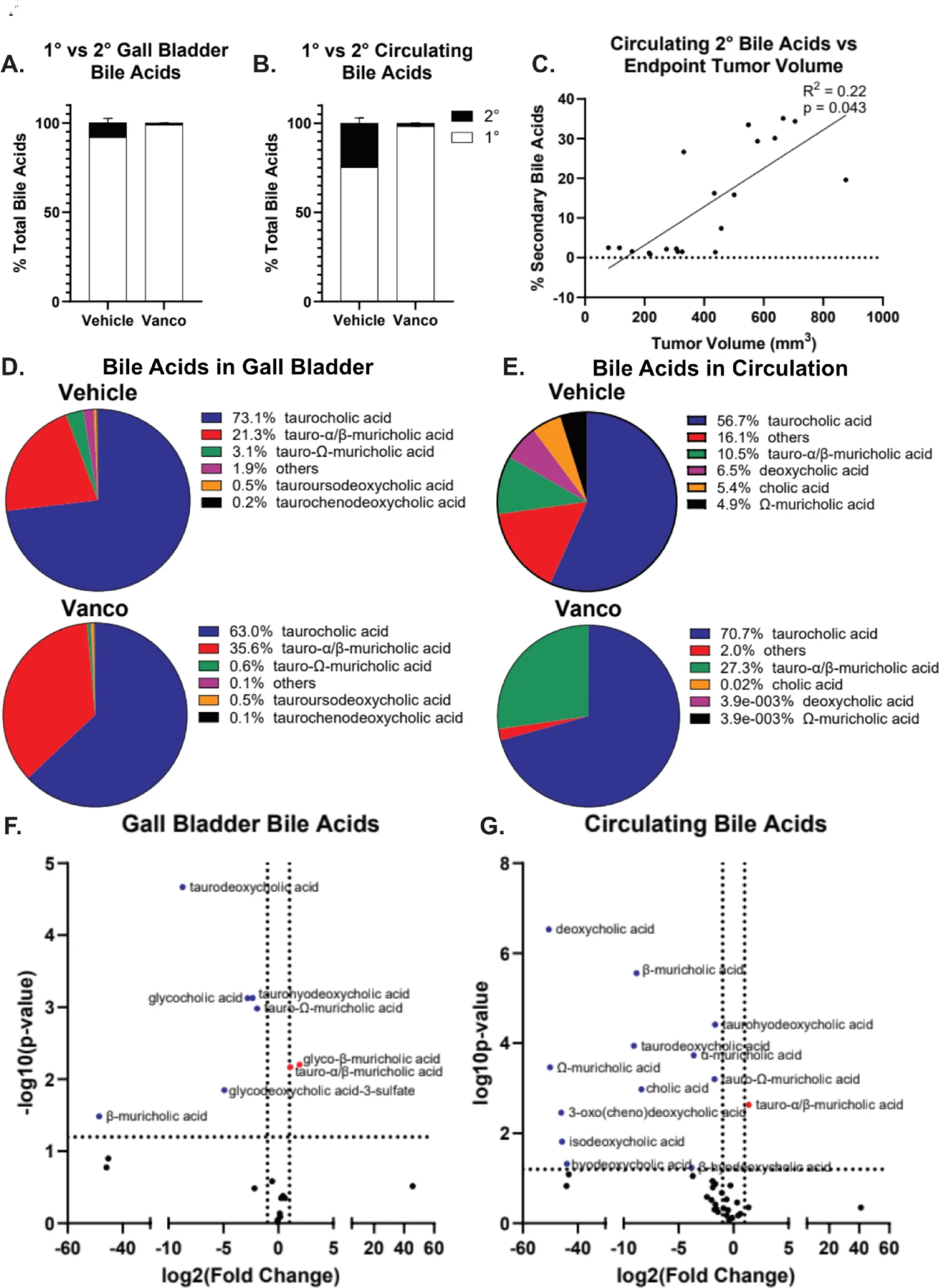
Vancomycin reduced secondary bile acids in the gall bladder and circulation. (**A-B**) Percent of primary and secondary bile acids in the gall bladder (**A**) and circulation (**B**) were measured. (**C**) Percent of secondary bile acids out of total in circulation is positively correlated with endpoint tumor volume by MaAsLin2 analysis, R^2^= 0.22, p = 0.043, N = 20. (**D-E**) The top 5 most abundant normalized circulating bile acid levels presented as parts of a whole pie charts with all remaining lower abundance bile acids summed up into “others” category in vehicle and vancomycin groups in the gall bladder (**D**) and circulating (**E**) bile acid pools. (**F-G**) Volcano plots of significant differentially abundant bile acids in the gall bladder (**F**) and circulation (**G**) with log_2_ fold change of greater than 1.5 and adjusted p value of less than 0.05 indicated by dot color, with red significantly elevated and blue significantly downregulated.

Because *Clostridium Cluster XIVa* regulates several metabolic pathways that impact MDMs, metabolomic profiling was performed on fasted bile and plasma to determine the impacts of vancomycin treatment on MDMs. MDMs measured included bile acids, short chain fatty acids, and amino acids with 67 metabolites quantified (*data not shown*). Vancomycin preconditioning did not significantly alter total bile acids (*data not* *shown*). Vancomycin significantly associated with reduced secondary bile acid levels in both the gallbladder and in circulation (Fig. 3A-B). The observed reduction in circulating secondary bile acids correlated negatively with endpoint tumor burden (Fig. 3C).

Furthermore, vancomycin preconditioning associated with reduced diversity of both circulating and gall bladder-associated bile acid pools (Figs. 3D). Simplified bile acid profiles are shown for both circulating and gall bladder-associated profiles by plotting the top five most abundant bile acids in each group and combining the more rare bile acids into a single category as “others.” Vancomycin-treated mice exhibited ablation of many primary and secondary bile acids in both the bile and plasma (Figs. 3F). Thus, metabolomic analysis suggested a potential role for specific bile acids in worsened tumor outcomes, which was reversed with vancomycin preconditioning of the gut microbiome.

**Fig. 4:**
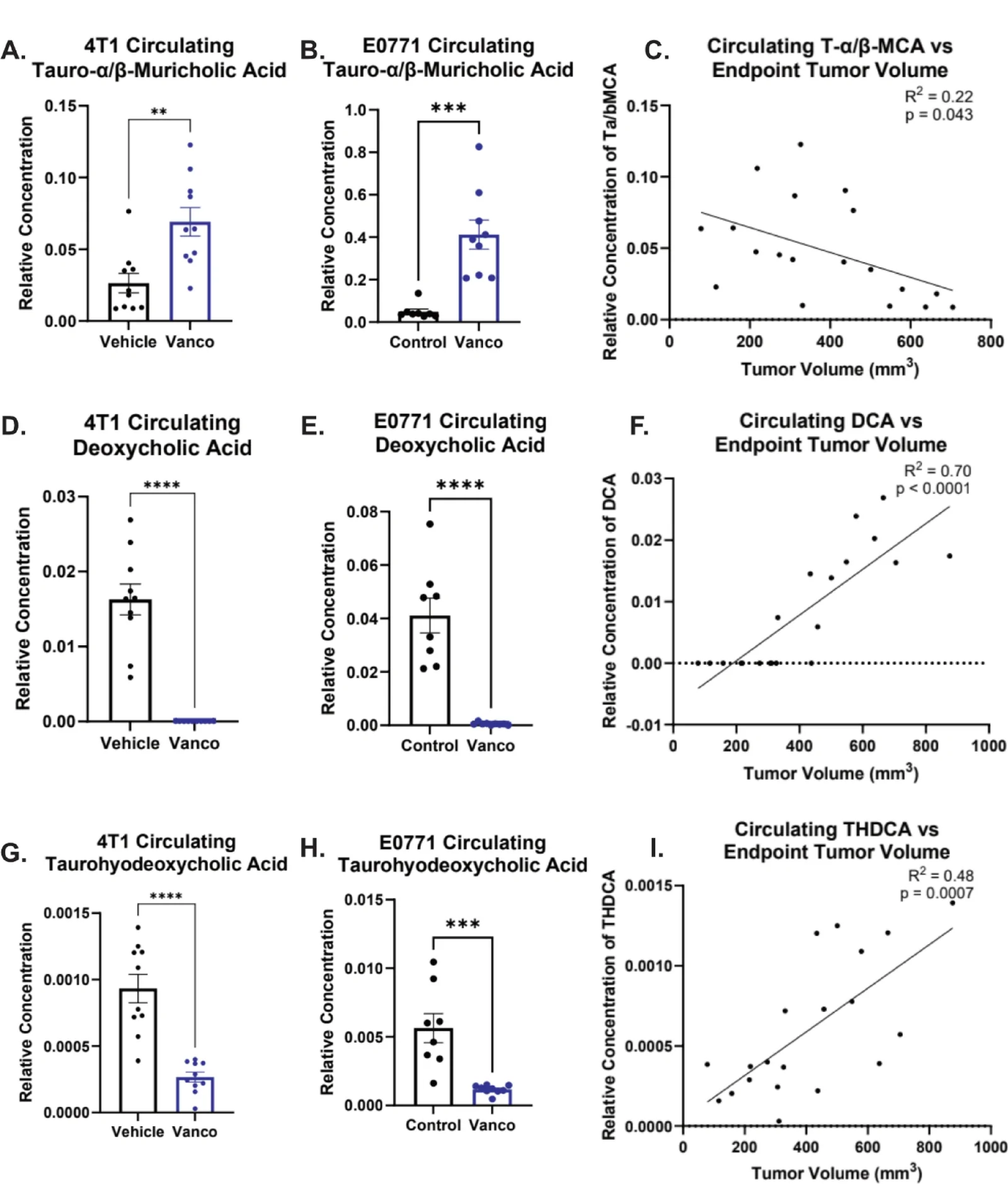
Candidate bile acids were associated with modulation of tumor outcomes. (**A-B**) Relative concentrations of circulating tauro-α/β-muricholic acid in the 4T1 (**A**) and E0771 (**B**) models presented as mean ± SEM with Student’s *t-*test for N = 7-10 per group. (**C**) Circulating tauro-α/β-muricholic acid levels in the 4T1 model correlated negatively with endpoint tumor volume by MaAsLin2 analysis, R^2^= 0.22, p = 0.043, N = 20. (**D-E**) Relative concentrations of circulating deoxycholic acid in the 4T1 (**D**) and E0771 (**E**) models presented as mean ± SEM with Student’s *t-*test for N = 7-10 per group. (**F**) Circulating deoxycholic acid levels in the 4T1 model correlated positively with endpoint tumor volume by MaAsLin2 analysis, R^2^= 0.70, p < 0.0001, N = 20. (**G-H**) Relative concentrations of circulating taurohyodeoxycholic acid in the 4T1 (**G**) and E0771 (**H**) models presented as mean ± SEM with Student’s *t-*test for N = 7-10 per group. (**I**) Circulating taurohyodeoxycholic acid levels in the 4T1 model correlated positively with endpoint tumor volume by MaAsLin2 analysis, R^2^= 0.48, p = 0.0007, N = 20. ***p < 0.001, ****p < 0.0001.

To identify potential candidate drivers of improved cancer outcomes induced by vancomycin treatment, circulating bile acids were evaluated for changes in relative concentration. Circulating tauro-α/β-MCA is a primary bile acid that was uniquely elevated following vancomycin preconditioning in both E0771 and 4T1 models when compared to vehicle-treated controls (Figs. 4A). Interestingly, tauro-α/β-MCA was also elevated in the gall bladder of these mice (Fig. 3F). Tauro-α/β-MCA relative abundance negatively correlated with 4T1 tumor volume (Fig. 4C).

Conversely, deoxycholic acid (DCA) and taurohyodeoxycholic acid (THDCA) are secondary bile acids. DCA and THDCA were significantly reduced in the plasma of vancomycin-conditioned mice compared to controls in both 4T1 and E0771 models (Figs. 4D-E, G-H). Circulating DCA and THDCA significantly positively correlated with tumor volume at endpoint (Figs. 4F, I). Bile acids modulate host immune signaling^14^. Therefore, we next tested the impact of vancomycin preconditioning on the systemic immune response and tumor immune microenvironment by flow cytometric analysis.

### Vancomycin preconditioning associated with elevated immune cells

**Fig. 5:**
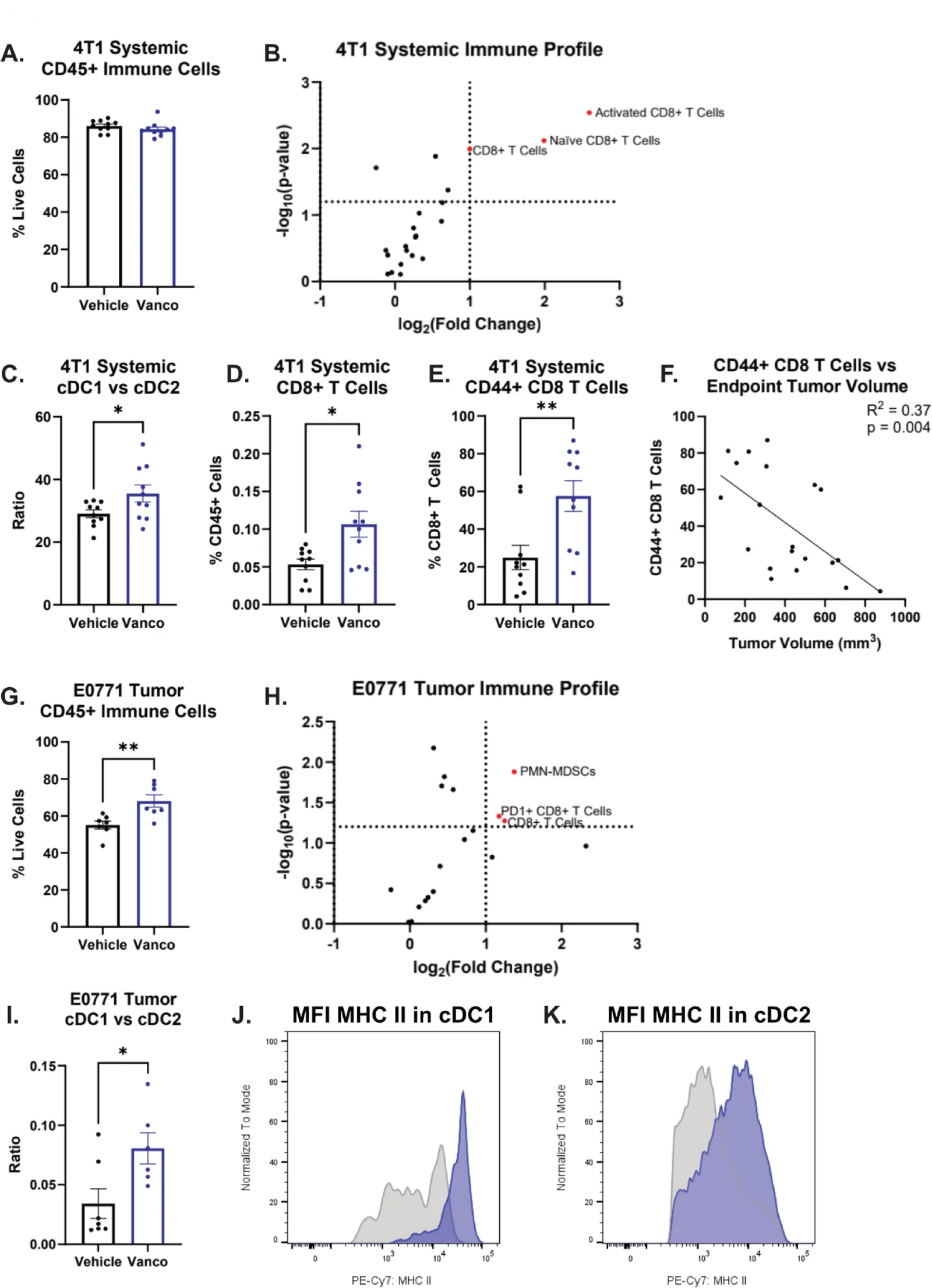
Vancomycin elevated key immune cells. (**A-E**) Flow cytometric analysis of 4T1 model spleens from BALB/cJ mice. (**A**) CD45+ total immune cells presented as mean ± SEM with Student’s *t-*test for N = 10 per group. (**B**) Volcano plot of significant differentially abundant immune subsets with log_2_ fold change of greater than 1.5 and adjusted p value of less than 0.05 indicated by dot color, with red significantly elevated and blue significantly downregulated. (**C-E**) Classical dendritic cell (DC) ratio of cDC1 (CD11c+, CD103+, CD11b-) to cDC2 (CD11c+, CD103-, CD11b+, **C**) cells, CD8+ T cells (**D**), and activated T cells (CD8+, CD44+, **E**) was quantified. Data are presented as mean ± SEM with Student’s *t-*test for N = 10 mice per group. (**F**) Activated T cells in the spleen correlated negatively with endpoint 4T1 tumor volume by linear regression analysis, R^2^= 0.37, p = 0.004, N = 20. (**G-K**) Flow cytometric analysis of E0771 tumor immune content from C57BL/6J mice. (**G**) CD45+ total immune cells presented as mean ± SEM with Student’s *t-*test for N = 7-8 per group. Outlier analysis removed 1 sample due to low cell count; this mouse was removed from all subsequent analysis. (**H**) Volcano plot of significant differentially abundant tumor immune subsets with log_2_ fold change of greater than 1.5 and adjusted p value of less than 0.05 indicated by dot color, with red significantly elevated and blue significantly downregulated. (**I**) Classical dendritic cell ratio of cDC1 (CD11c+, CD103+, CD11b-) to cDC2 (CD11c+, CD103-, CD11b+) cells. (**J-K**) Mean fluorescent intensity of MHC II in cDC1 cells (**J**) and cDC2 cells (**K**) was quantified. Data are presented as histograms normalized to mode with MFI plotted as median peak fluorescent intensity for representative samples of each group. Vehicle is shown in grey, and vancomycin is shown in blue. *p < 0.05, **p < 0.01.

To examine vancomycin-associated effects on systemic immunity, flow cytometric analysis was conducted on spleens from the 4T1 model with 19 markers to identify 28 cell immune subsets. Although overall CD45 + immune cell frequency was not impacted (Fig. 5A), spleens demonstrated enrichment of various CD8 + T cell subsets including total, naïve, and activated CD8 + T cells (Fig. 5B). No immune cell subsets were downregulated in the spleen. Furthermore, a significant increase in the classical dendritic cell (cDC) 1 vs. cDC2 ratio with vancomycin preconditioning indicated increased content of cDCs responsible for cross-presentation to CD8+ T cells (Fig. 5C). CD8 + T cells were elevated within the spleen, including the CD44+ activated subset (Figs. 5B, D-E). Activated CD8+ T cells correlated negatively with 4T1 tumor volume at endpoint (Fig. 5F).

To next determine the extent of impact of vancomycin preconditioning on the immune response in the tumor microenvironment (TME), E0771 BC tumors were analyzed by flow cytometry. Tumor immune cell infiltration was significantly increased in vancomycin-conditioned mice compared to controls (Fig. 5G). Interestingly, no cell types were significantly reduced by vancomycin in the TME either (Fig. 5H). CD8+ T cell content as well as the PD-1+ subset of CD8+ T cells was elevated in the E0771 TME (Fig. 5H). Interestingly, polymorphonuclear myeloid derived suppressor cells (PMN-MDSCs) were also elevated after vancomycin treatment (Fig. 5H), cell types that are associated with immunosuppression^30^. Elevated cDC1 vs. cDC2 ratio within E0771 tumors supports an increased capacity for cross-presentation to CD8+ T cells across both BC models (Fig. 5I). Elevated MHC class II in both cDC subsets suggests increased antigen presentation capacity in tumors from mice treated with vancomycin compared to controls (Figs. 5J-K). Taken together, immune phenotyping across two models suggests that vancomycin preconditioning of the gut microbiome associated with both systemic immune modulation and changes to the TME that may support antitumor immunity and blunt primary tumor progression.

### Vancomycin preconditioning associated with reduced BC lung colonization in an experimental lung colonization model

**Fig. 6:**
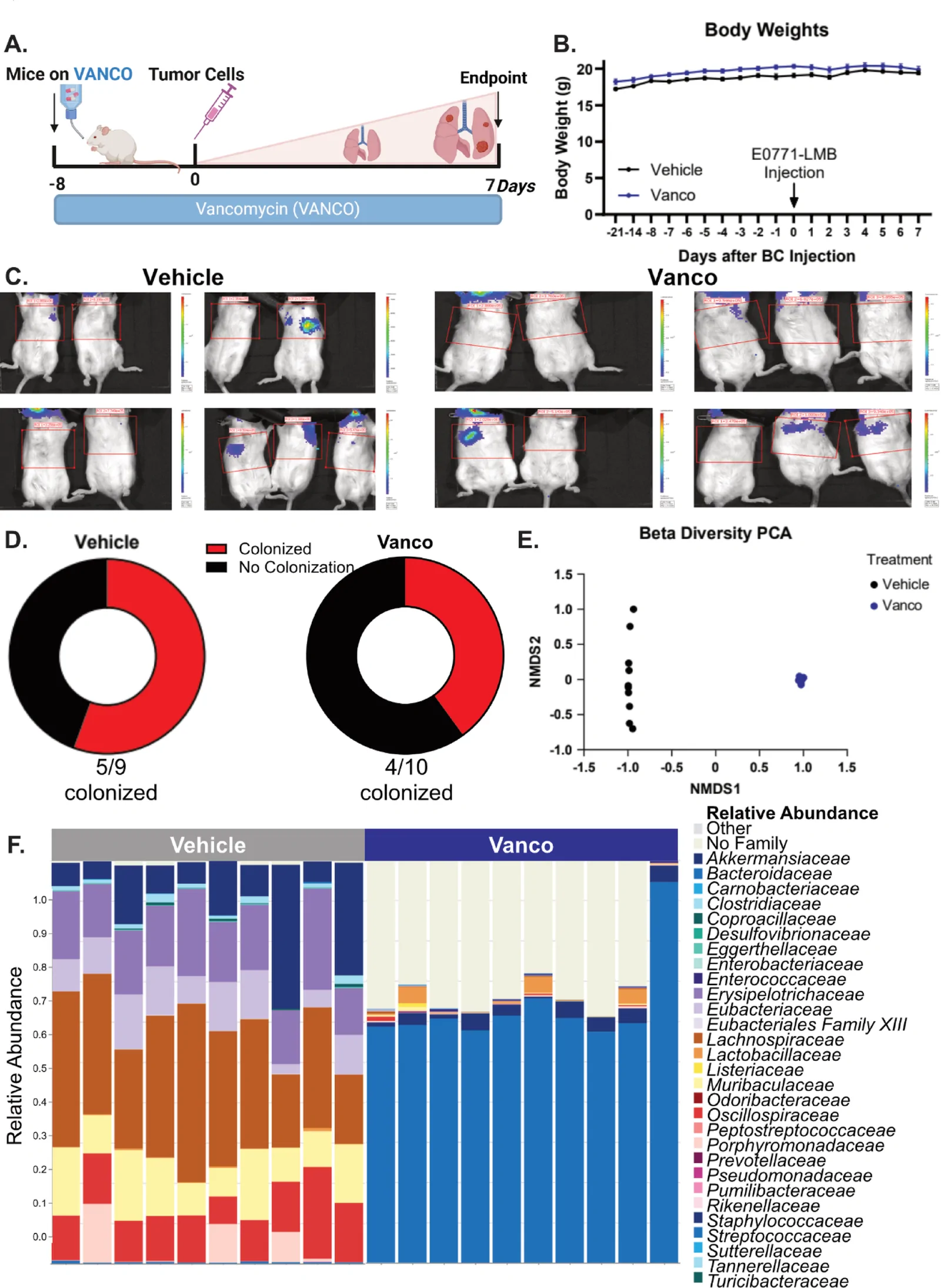
Vancomycin reduced breast cancer E0771.LMB lung colonization model. (**A**) E0771.LMB Study Design: Female B6(Cg)-*Tyr*^c-2J^/J mice (N = 9-10 per group) were placed on water containing vancomycin (1 g/L) or plain sterile water for 8 days. E0771-LMB breast cancer cells were injected into the retro-orbital vein. (**B**) Body weights were measured for duration of study with data presented as mean ± SEM with multi-effects analysis and Fisher’s LSD correction. (**C**) Tumor cell burden in the lung was measured at endpoint by IVIS live imaging. (**D**) The number of mice with quantifiable lung colonies was enumerated out of total mice. (**E**) Microbial beta diversity of cecal contents collected from E0771 model at endpoint is shown by principal component analysis of metagenomic sequencing with clustering determined by Bray-Curtis distances. (**F**) Relative abundance of microbial families is reported in cecal contents harvested at study endpoint. Study cartoon was created with BioRender.

Vancomycin preconditioning of the gut microbiome associating with changes in bile acids and specific immune cells prompted us to next investigate BC lung colonization in an experimental lung colonization model due to the high lethality of metastatic disease^31^. Even with recent advances in BC treatment, metastatic BC has a 5-year survival rate of just 32%. Female albino B6 mice were preconditioned with vancomycin or control water as described in study design (Fig. 6A). The use of albino mice allowed for bioluminescent imaging and quantification of lung colonization without interference from C57BL/6J fur. Following 8 days of vancomycin treatment, E0771-lung metastasis branch (LMB) cells that preferentially colonize the lungs were injected via retro-orbital vein as an experimental model of BC lung colonization. Vancomycin exposure did not impact body weight or water intake (*data not shown*) for the entire study duration (Fig. 6B).

At endpoint, lung colonization was measured using IVIS luminescence imaging, which did not differ between vehicle or vancomycin treatments (Fig. 6C). However, when the number of mice displaying lung colonization masses was quantified, fewer vancomycin-treated mice had quantifiable masses by bioluminescent imaging at endpoint when compared to the control group (Fig. 6D). Indeed, 55.6% of control mice had quantifiable lung colonization at endpoint while only 40% of vancomycin-treated mice exhibited lung colonization. Thus, vancomycin preconditioning associated with reduced BC colonization to the lungs; however, once in the lung, tumor growth was not different between vancomycin or control groups.

Similar to the primary tumor models, the cecal microbiota was significantly impacted by vancomycin preconditioning. PCA clustering demonstrated significant differences in cecal content microbiome composition and Beta diversity at study endpoint as in both the 4T1 and E0771 models (Fig. 6E). The cecal contents of vancomycin-conditioned mice were dominated by family *Staphylococcaceae* (Fig. 6F), similar to the primary tumor model (Supplemental Fig. 5A).

## Discussion

The gut microbiome impacts cancer risk and outcomes, including responses to therapy^3,4,6,11,32–34^. Herein, we investigated the role of bile acids in BC progression and lung colonization. Briefly, bile acid pools include primary, secondary, and tertiary, which can be conjugated to an amino acid (glycine, taurine, other), deconjugated, or have biochemical modifications^14^. Primary bile acid signaling appears to be more pro-immune in some tissues, while secondary bile acid signaling may be more immunosuppressive and lead to impaired cancer outcomes^16,18,19^; however, this is an emerging field with immense growth in recent years. Bile acid pool composition is modulated by the gut microbiome, especially *Clostridium Cluster XIVa*. Members of this cluster of microbes, comprised of eight genera, express the 7-alpha-dehydroxylase enzyme responsible for the rate-limiting deconjugation step of primary to secondary bile acid conversion^21,35–39^. Therefore, we aimed to test if preconditioning the gut microbiome by targeting bacteria that impact the production of secondary bile acids by using a gut-impermeable antibiotic would reduce BC tumor progression and lung colonization.

We demonstrated that vancomycin preconditioning reshaped the gut microbiome and associated with shifted bile acid pools in both the gall bladder and circulation as expected. Although antibiotic-mediated microbiome dysbiosis may be a driver of poor BC outcomes in certain contexts, previous work supports the idea of using antibiotics targeting specific groups of bacteria to manipulate the gut microbiome composition to improve tumor outcomes and response to therapies^40,41^. Indeed, we report that vancomycin-mediated ablation of the commensal gut microbiome resulted in host priming that associated with improved outcomes across several murine models of BC. BC progression was significantly improved with delayed tumor onset, slowed progression, and reduced endpoint tumor burden, with evidence of increased regression and reduced colonization to the lung following vancomycin preconditioning. 

Analysis of cecal contents revealed reduced microbial diversity after vancomycin preconditioning and significant variations to beta diversity metrics. At the family level, vancomycin also depleted taxa such as *Lachnospiraceae*,* Eubacteriaceae*,* Oscillospiraceae*, and *Lactobacillaceae*, which are groups that are associated with worsened cancer outcomes^33,42–44^, while vancomycin increased the relative abundance of potentially anti-tumor microbes such as *Akkermansiaceae* and *Paenibacillaceae*^25–28^. One particular species elevated by vancomycin treatment, *P. ginsengihumi*, is an acetate-producing species associated with reduced proliferation of cancer cells^25^. Although acetate was reduced in circulation (*data not shown*), local TME content may be more relevant to BC progression and immune responses as acetate can be used as a fuel source by tumor-infiltrating lymphocytes^45^. As expected with vancomycin treatment based on Ma et *al*.^18^, a significantly decreased relative abundance in members of *Clostridium Cluster XIVa* was consistent across models.

Functional pathway analysis revealed downregulation of microbial fermentation and biosynthetic programs that may otherwise contribute to metabolic stress. Microbial fermentation produces short-chain fatty acids such as butyrate, acetate, and propionate, and other metabolites, including lactate, succinate, and secondary bile acids. These metabolites alter TME metabolism and inflammation which can advance cancer progression^46^. Also reduced by vancomycin preconditioning were pathways associated with coenzyme A production, a precursor of vitamin B5 production^47^. Vitamin B5, also known as pantothenic acid, is a known driver of MYC oncogenic metabolism, which leads to enhanced tumor progression in BC patients and preclinical models^48^. These findings present an additional potential mechanism underscoring the beneficial role of vancomycin-induced microbiome priming in BC outcomes.

Microbially-associated metabolomic analysis of bile and plasma samples revealed significant shifts in both gall bladder-associated and circulating bile acid pools. Vancomycin associated with reductions in both circulating and gall bladder secondary bile acids while primary bile acids were correspondingly elevated. The observed reduction in circulating secondary bile acids correlated negatively with endpoint tumor burden, supporting the idea that blocking secondary bile acid production may improve BC outcomes. Thus, vancomycin preconditioning ablated microbiota necessary for primary to secondary bile acid conversion in our model, as expected based on similar study designs^18^. The near-complete loss of *Clostridium Cluster XIVa* underscored a potential link between antibiotic targeting of the gut microbiome and bile acid composition remodeling, which was supported by analysis of stored and circulating bile acid pools. Taken together, these data support the central hypothesis that lower secondary bile acid production in combination with higher primary bile acid levels potentially associated with an anti-tumor immune environment. Obesity and metabolic dysfunction are associated with worsened BC risk and outcomes; metabolic dysfunction is also related to microbiome dysbiosis as well as an increase in systemic secondary bile acids due to alterations in the gut microbiome, specifically an increase in abundance of *Clostridium Cluster XIVa*^14,49,50^. As this work is largely correlative, future studies are required beyond the scope of this manuscript examining the contribution of *Clostridium Cluster XIVa* or microbes therein to determine the direct impact of 7-alpha-dehydroxylase enzyme activity on cancer progression as well as to elucidate the mechanistic role of vancomycin preconditioning in the observed improvements in BC outcomes.

Findings presented herein align with earlier work published by our group and others that demonstrate bile acid signaling, particularly through the bile acid nuclear hormone receptor FXR, may restrain BC growth and improve survival by altering T cell function^21,51^. Our group has further demonstrated that FXR activation through a small molecule pharmaceutical bile acid memetic obeticholic acid (OCA, INT-747, known commercially as Ocaliva) slowed BC progression in vivo and inhibited cancer cell progression and migration in vitro^21^. Furthermore, high FXR expression in tumors from patients correlated with improved survival in estrogen receptor negative and basal-like BC, the most aggressive subtypes of BC^21^. Others have also shown that FXR signaling is anti-tumorigenic in pre-clinical studies^35–39^. Lastly, Ma et *al.* demonstrated that shifting the bile acid pools via vancomycin treatment blunted tumor growth and improved anti-tumor immune responses in multiple types of cancer, including hepatocellular carcinoma^18^.

In both 4T1 and E0771 BC models, we report that circulating DCA and THDCA correlated positively with tumor volume and were significantly reduced with vancomycin exposure, which points to indirect, potential immune-mediated effects to be tested in future studies. In contrast, the primary bile acid tauro-α/β-MCA increased after vancomycin preconditioning in both models. Although tauro-α/β-MCA is a known FXR antagonist^52^, elevated levels of this bile acid may signal through another bile acid receptor TGR5 (*Gpbar1*), which has been shown to have the potential to enhance immune function^53^. Tumor and systemic immune cell profiling revealed that vancomycin preconditioning associated with cells that may support enhanced anti-tumor immunity, with tumor-infiltrating CD45+ immune cells and CD8+ T cells, increased, indicating a heightened cytotoxic T cell response. Conversely, we observed elevations of PMN-MDSCs in the spleen as well as PD-1+ CD8+ T cells in the TME, which may be an indicator of either immune activation or exhaustion^30,54,55^. These findings underscore that the systemic and local immune milieu is complex and additional studies are required to fully understand the relationship between vancomycin preconditioning and the potential for improved mediators associated with anti-tumor immunity. Elevated cDC1 to cDC2 ratios and higher MHC-II expression in DC subsets further suggest improved antigen presentation capacity to CD8+ T cells and stronger immune cell responsiveness in two mouse strains with differing immune contexts, though this data is correlative and requires confirmation with gain and loss of function studies. Taken together, these observations suggest that vancomycin preconditioning triggers microbially-mediated immune reprogramming through a microbiome–bile acid–dendritic cell axis that may support the potential for T cell–mediated cytotoxic immune responses. These findings strengthen the rationale for targeting bile acid pathways as a therapeutic angle in BC, especially given the growing data that demonstrate MDMs are able to shape T cell metabolism and effector functions, DC maturation and antigen presentation, and anti-tumor immune responses following immunotherapy, though immunotherapy effectiveness was not specifically tested in this study^6,11,^^56,57,13^.

There are some limitations to this study. Antibiotic treatment is a blunt tool and cannot distinguish between the effects of specific taxa that were more susceptible to antibiotics compared with broad ecological disruption, off-target immune modulation, and indirect metabolic effects. Furthermore, preclinical and clinical literature has demonstrated that broad-spectrum antibiotic exposure is frequently associated with impaired response to immune checkpoint blockade and poorer outcomes in cancer patients, particularly in the context of PD-1/PD-L1-based immunotherapy^58–64^. It is likely that differences in antibiotic class, timing, duration of exposure, tumor type, host metabolic status, and treatment context may contribute to divergent effects across studies. Therefore, more targeted approaches will be required to elucidate the mechanistic underpinnings of these findings. Also, while associations between individual bile acids and tumor outcomes were significant and strong, causal pathways need further testing using gain- and loss-of-function approaches to confirm the mechanism of these interactions and their specific impacts on the BC TME. Despite these limitations, our data collectively show that vancomycin disrupts secondary bile acid production, which ultimately slowed both primary tumor onset as well as progression and tumor cell colonization of the lung. Overall, this study highlights a potential microbiome–bile acid–immune system interaction that may be leveraged to improve BC outcomes and points toward novel translational strategies that influence microbial metabolism in an effort to potentially shape anti-tumor immunity with the goal to improve BC outcomes.

## Materials and methods

### Reagents

All reagents were obtained from Sigma-Aldrich (St. Louis, MO) unless otherwise noted. Fetal bovine serum (FBS, Gibco, Waltham, MA), 100x GlutaMAX (Gibco), and 100x Penicillin/Streptomycin (HyClone, Pittsburgh, PA) were purchased from ThermoFisher (Waltham, MA). RPMI 1640 and Matrigel were from Corning (Tewksbury, MA). Vancomycin (V2002) was purchased from Sigma-Aldrich. Flow cytometry antibodies, compensation beads, and reagents were from Tonbo Biosciences, Inc. (San Diego, CA), and Biolegend (San Diego, CA), as described in Supplemental Table 1.

### Animals

Murine studies were performed with approval and in accordance with the guidelines of the Institutional Animal Care and Use Committee (IACUC) at the University of Tennessee Health Science Center and in accordance with the National Institutes of Health Guide for the Care and Use of Laboratory Animals. All animals were housed in a temperature-controlled facility with a 12-hour light/dark cycle and *ad libitum* access to rodent chow (7912 Teklad, Indianapolis, IN). Six-week-old female specific pathogen-free BALB/cJ mice (Jackson stock number: 000651) and C57BL/6J mice (Jackson stock number: 000664) were purchased from Jackson Laboratories (Bar Harbor, ME). Mice were randomized into 5 mice per cage. Mice were housed for 2 weeks prior to antibiotic (ABX) intervention to normalize baseline microbiomes. Mice were either assigned to plain water vehicle control or vancomycin ABX intervention groups (*N* = 10 mice per group). Mice in the Vancomycin group received vancomycin (1 g/L) in their drinking water. Water bottles were changed every 7 days, and water intake was daily measured by weight to confirm consistency of antibiotic exposure. Solubility of antibiotic in water was confirmed between water bottle replacements. Previous work has shown that vancomycin is stable in solution for up to 30 days at room temperature^65^. All water was autoclaved prior to use, and ABX water was prepared under sterile conditions. Body weights were measured daily.

### Cell culture and tumor cell implantation

4T1 murine adenocarcinoma BC cells, purchased from ATCC (CRL-2539), or E0771 murine adenocarcinoma BC cells expressing luciferase, a gift from Hasan Korkaya (Wayne State University, Detroit, MI), were cultured as previously described^11,30,34,66^. Cells tested negative for *Mycoplasma* (LT07-318 Lonza, Walkersville, MD). Briefly, cells were cultured in RPMI 1640 containing 10% FBS, 2 mM GlutaMAX, 100 UI/mL penicillin, and 100 µg/mL streptomycin in a humidified chamber at 37 °C under 5% CO_2_. 4T1 cells were orthotopically injected in the left fourth mammary fat pad of 8-week-old mice at 450,000 cells in 100 µL of 25% Matrigel Matrix High Concentration (354248 Corning), 75% RPMI with a final Matrigel concentration of 5 mg/mL. E0771 cells were orthotopically injected into the left fourth mammary fat pad of 8-week-old mice at 250,000 cells in 100 µL of 25% Matrigel Matrix High Concentration (354248 Corning), 75% RPMI with a final Matrigel concentration of 5 mg/mL. Starting seven days after implantation, tumor growth was monitored every third day by measuring the length and width of the tumor using digital calipers. Investigators were blinded to treatment group during tumor measurements. Tumor volume was calculated using the following formula: Volume = (width)^2^ × (length)/2^66^. No tumors failed to take in any of the studies. The number of mice with regressed tumors compared to total palpable tumors at endpoint was recorded using 50% tumor regression in accordance with previous work^67^. At experimental endpoint (day 13 or 21 after tumor cell injection), excised tumor mass was determined.

### Lung colonization model

Six-week-old female specific pathogen-free B6(Cg)-Tyr^c-2 J^/J (albino C57BL/6J) mice (Jackson stock number: 000058) were purchased from The Jackson Laboratories and treated as above. Mice were either assigned to water vehicle control or ABX intervention groups (*N* = 9–10 mice per group). Mice in the ABX group received vancomycin (1 g/L) in their drinking water. Water bottles were changed every 7 days, and water intake was measured daily by weight. All water was autoclaved prior to use, and ABX water was prepared under sterile conditions. Body weights were measured daily.

E0771-LMB (or E0771.LMB for lung metastasis branch, ATCC CRL-3405) murine adenocarcinoma BC cell expressing luciferase, a gift from Chad Pecot (University of North Carolina, Chapel Hill, NC), were cultured as previously described^68^. This is a commonly used approach for an in vivo model of metastasis^69–70^, herein referred to as lung colonization. Cells tested negative for *Mycoplasma* (LT07-318 Lonza). Briefly, cells were cultured in RPMI 1640 containing 10% FBS, 2 mM GlutaMAX, 100 UI/mL penicillin, and 100 µg/mL streptomycin in a humidified chamber at 37 °C under 5% CO_2_. E0771-LMB cells were injected into the retro-orbital vein of 8-week-old mice at 100,000 cells in 50 µL of PBS (10010-023 Gibco). Tumor cell burden in the lung was measured via bioluminescence using the In Vivo Imaging System (IVIS). Images and measurements of bioluminescent signals were acquired and analyzed using the Living Image software version 123. Investigators were blinded to treatment group during analysis. D-luciferin was dissolved in sterile PBS (0.015 g/mL) and injected intraperitoneally at 150 mg/kg 15 min prior to imaging. Image acquisition occurred over 1 min. Identically sized regions of interest (ROI) were drawn around lung sites and quantified as radiance (photons/second/cm^2^/sr). Background bioluminescence was measured and subtracted from reported measurements.

### Tissue and blood collection

At study endpoint, mice were fasted 4 h prior to isoflurane euthanasia. Blood was collected via cardiac puncture into EDTA-coated syringes as a secondary method of euthanasia. Plasma was separated from other blood components by centrifugation at 1200 x g for 45 min at 10 °C. Bile was collected from the gall bladder and diluted 1:1000 in PBS. Mammary tumors, un-injected mammary fat pads from contralateral side (MgAT), gonadal adipose (GAT), tumor-adjacent adipose tissue (TAAT), cecal contents, spleen, and liver were weighed and either snap frozen in liquid nitrogen, placed in a cassette for 24-hour fixation in 10% formalin, or digested into a single cell suspension for flow cytometry. All frozen samples were stored at −80 °C until analyzed.

### Flow cytometric analysis of spleens and tumors

Spleen single cell suspensions were obtained by grinding spleens against a 70 μm filter using a syringe plunger. A portion of the excised tumors (~ 100 mg) were minced using scissors in RPMI media containing enzyme cocktail mix from Miltenyi Biotec mouse tumor dissociation kit (130-096-730 Miltenyi Biotec, Auburn, CA). Tumor pieces were further digested as per manufacturer’s instructions, and digested tissue was filtered through 70 μm strainer to obtain a single cell suspension. Antibodies used (Supplemental Table 1) were titrated and the separation index was calculated using FlowJo version 10 software. Following red blood cell lysis (420301 BioLegend), viability was determined by staining with LD Fix Blue dye (L34962 Thermo Fisher) or Ghost dye (TNB-13–0870 Tonbo Biosciences, Inc.) followed by FcR-blocking (TNB-70–0161-M001 Tonbo Biosciences, Inc.). Cells were stained with fluorescently labeled antibodies and fixed in Perm/fix buffer (TNB-1020-L050 Tonbo Biosciences Inc.). Stained cells were analyzed using a Cytek Aurora Cell Sorter for splenic analysis and Bio-Rad ZE5 flow cytometer for tumor immune microenvironment at the UTHSC Flow Core facility. Fluorescence minus one (FMO) stained cells and single color Ultracomp Beads (01–2222-42 Invitrogen, Carlsbad, CA) or splenocytes were used as negative and positive controls, respectively. Data were analyzed using FlowJo version 10 software, and investigators were blinded to treatment group during analysis. Gating schemata are shown for both 4T1-BALB/cJ spleens and E0771-C57BL/6J tumors, respectively (Supplemental Figs. 1–2).

### Metagenomic whole-genome sequencing analysis

Cecal contents from the 4T1-BALB/cJ model were collected at the time of sacrifice, flash-frozen, and stored at −80 °C until analysis. A subset of frozen cecal contents, 50 mg, was transferred into a sample collection tube containing DNA stabilization buffer (Transnetyx Inc., Cordova, TN) to ensure reproducibility, stability, and traceability. Reproducible DNA extraction was automated and performed by Transnetyx. In brief, per Transnetyx processes, the Qiagen DNeasy 96 PowerSoil Pro kit was used for extraction, the KAPA HyperPlus kit was used for library preparation, and the Illumina 2 × 150-bp sequencing kits were used on a NextSeq 2000^71^. Unique dual-indexed adapters were used to ensure that reads and/or organisms were assigned correctly.

After quality control review, libraries were sequenced using shotgun sequencing at a depth of 2 million 2 × 150-bp read pairs. Environmental controls included empty tubes passed through an identical collection, extraction, and amplification process, and controls remained free of contaminating DNA amplification (*data not shown*). Sequencing data were uploaded into One Codex online platform and aligned to the software database consisting of > 127 K whole microbial reference genomes (One Codex; One Codex Inc., San Francisco, CA)^72^. Alpha diversity via Shannon and Simpson indices as well as principal component analysis (PCA) using Bray-Curtis distances were performed in One Codex. Microbiome compositions of individual mice were examined for evidence of cage effects as in our previous publications^11,73^, and none were observed. Functional analyses were analyzed using the Humann3 plug in on One Codex and graphed as fold change between vancomycin and vehicle control groups^29^. Selected functional pathways significantly impacted by vancomycin treatment were identified by the MetaCyc database. MaAsLin2 was used for correlation analyses with tumor outcomes and metabolomic data^74^.

### 16 S microbiome analysis

Cecal contents from the E0771-C57BL/6J model were collected at the time of sacrifice, flash-frozen, and stored at −80 °C for analysis. For DNA isolation, samples were placed in 1 mL extraction buffer [50 mM Tris (pH 7.4), 100 mM EDTA (pH 8.0), 400 mM NaCl, 0.5% SDS] containing 20 µL proteinase K (20 mg/mL). Approximately 250 µL of 0.1-mm-diameter zirconia/silica beads (BioSpec Products) was added to extraction tubes and mechanically perturbed using a Mini-Beadbeater-8 cell disrupter (BioSpec Products) for 2 × 1 min. Tubes were incubated at 55 °C overnight with agitation. DNA was extracted with the phenol: chloroform: isoamyl alcohol method, precipitated with ethanol, and dissolved in nuclease-free water. Purified DNA samples underwent 16 S library preparation and NextGen Illumina MiSeq sequencing at Novogene Bioinformatics Technology Co (Davis, CA). Environmental controls included empty tubes passed through an identical collection, extraction, and amplification process, and controls remained free of contaminating DNA amplification (*data not shown*).

Paired-end reads were processed in R studio version 4.3.2 using the DADA2 pipeline^75^ to correct sequencing errors and to determine amplicon sequence variants (ASV) as has been previously published^11^. ASVs were assigned taxonomy by comparison to Ribosomal Database Project release 18.0^76^. Differential abundance of individual taxa was assessed using Corncob^77^, while alpha diversity and tSNE analysis were conducted using vegan^78^. Microbiome compositions of individual mice were examined for evidence of cage effects, and none were observed. MaAsLin2 was used for correlation analyses^74^, as above.

### Bile acid composition analysis

Plasma and bile collected at endpoint were analyzed for bile acid composition. Bile acid compositions were measured at the University of Chicago Duchossois Family Institute Host-Microbe Metabolomics Facility as previously described^11^. In brief, bile acid analyses were reported as relative and for a subset of bile acids, quantitative using standards as in previous publications^79–81^. Primary, secondary, and glyco/tauro-conjugated subclasses were measured using negative mode liquid chromatography-electrospray ionization-quadrupole time-of-flight-MS [(-)LC-ESI-QTOF-MS], on an Agilent 6546 LC/Q-TOF system. All compounds have been validated through retention time and fragmentation comparison to standards and available databases. Normalized peak areas were calculated by dividing raw peak areas of targeted analytes by averaged raw peak areas of internal standards. Volcano plots were created in GraphPad Prism version 10, and correlation analyses were conducted using MaAsLin2^74^ in R studio version 4.3.2 or linear regression in GraphPad Prism version 10.

### Statistical analyses

All analyses and figure generation were performed in R studio version 4.3.2 or GraphPad Prism version 10 (GraphPad Software, Inc., La Jolla, CA). Study designs and cartoons were generated in Biorender. Sample sizes were determined by power analysis calculations and pilot experiments. Statistical differences between experimental groups were determined using one-way or two-way ANOVA (as noted in figure legends) with Fisher’s LSD test for individual comparisons. Outliers were identified and excluded based on the ROUT method with Q = 1% in GraphPad Prism. For body weight, water intake, and tumor volume over time within animals, data was treated as repeated measures. All data are shown as mean ± standard error of the mean (SEM). Calculated p values < 0.05 were considered statistically significant.

### Study approval

All experimental protocols were approved by the Institutional Animal Care and Use Committee and Institutional Biosafety Committee at the University of Tennessee Health Science Center.

## Supplementary Information

Below is the link to the electronic supplementary material.

Supplementary Material 1

## Data Availability

The data generated in this study are available upon request from the corresponding author. Metagenomic whole genome shotgun and 16 S microbiome sequencing data were deposited into NCBI Short Read Archive database and are publicly available (NCBI BioProject ID PRJNA1419445, https://dataview.ncbi.nlm.nih.gov/object/PRJNA1419445). Any code written to analyze data is available upon request.
