# Supplementary material for: Reduction of secondary bile acids following microbiome pre-conditioning was associated with reduced breast cancer progression
Source: Sci Rep. 2026 Jul 31;16:30183. doi: 10.1038/s41598-026-59669-3 (PMC13623637; doi:10.1038/s41598-026-59669-3)
Supplement: Supplementary file 1 [file 41598_2026_59669_MOESM1_ESM.pdf]

Reduction of secondary bile acids through microbiome pre-conditioning blunted tumor progression and lung colonization associated with improved anti-tumor immunity in breast cancer.

Authors: Margaret S. Bohm[1], Johnathan R. Yarbro[2], Clàudia Font Torres[2], Zeid T. Mustafa[2], Mehdi Chaib[3], Tahliyah S. Mims[4], Sydney C. Joseph[2], Joel H. Elasy[2], Laura M. Sipe[5], Katherine L. Cook[6], Joseph F. Pierre[4], Liza Makowski[1,2,3,7*]

Running Title (60 characters): Vancomycin associated with improved outcomes in breast cancer

Supplemental Table and Figures

Supplementary Table 1. Antibodies for flow cytometric analysis of immune interactions.

Antibody	Source	Identifiers	Dilutions
RedFluor 710 anti-human/mouse CD11b	Tonbo	80-0112-U025	1:20
APC anti-mouse Ly-6C	BioLegend	128015	1:40
PE anti-mouse F4/80	Tonbo	50-4801-U025	1:40
PerCP-Cyanine5.5 anti-mouse Ly-6G	Tonbo	65-1276-U025	1:40
Brilliant Violet 650 anti-mouse CD11c	BioLegend	117339	1:40
PE/Cyanine7 anti-mouse I-A/I-E (MHC class II)	BioLegend	107630	1:40
FITC anti-mouse CD45	BioLegend	103108	1:40
Brilliant Violet 711 anti-mouse CD274 (B7- H1, PD-L1)	BioLegend	124319	1:10
APC/Cyanine7 anti-mouse CD103	BioLegend	121432	1:5
FITC anti-mouse CD8a	Tonbo	35-0081-U025	1:10
Brilliant Violet 605 anti-mouse CD4	BioLegend	100548	1:10
Brilliant Violet 785 anti-mouse CD3ε	BioLegend	100355	1:4
PE/Dazzle 594 anti-mouse NK-1.1	BioLegend	108747	1:10
Brilliant Violet 421 anti-mouse CD279 (PD-1)	BioLegend	109121	1:10
PerCP/Cyanine5.5 anti-mouse CD45	BioLegend	103132	1:20
PE anti-mouse/human CD44	BioLegend	103008	1:5
Brilliant Violet 650 anti-mouse CD69	BioLegend	104541	1:20
Alexa Fluor 700 anti-mouse CD62L	BioLegend	104426	1:40
BUV 395 anti-mouse CD11b	ThermoFisher	363-0112-82	1:20
BUV 563 anti-mouse CD8a	ThermoFisher	365-0081-82	1:5
Brilliant Violet 480 anti-mouse CD3e	ThermoFisher	414-0031-82	1:5
BUV 737 anti-mouse CD103	ThermoFisher	367-1031-82	1:10
cFluor UV 790 anti-mouse CD19	ThermoFisher	R7-21003	1:10
Super Bright 702 anti-mouse I-A/I-E (MHC class II)	ThermoFisher	67-5321-82	1:40
Super Bright 780 anti-mouse CD11c	ThermoFisher	78-0114-82	1:10
NovaFluor Yellow 700 anti-mouse CD274 (PD-L1)	ThermoFisher	M036T03Y06	1:5
APC-Cy7 anti-mouse CD44	ThermoFisher	25-0441-U100	1:40

Supplemental Figure 1.

4T1 Systemic Immune Profiling Flow Cytometry Gating Scheme

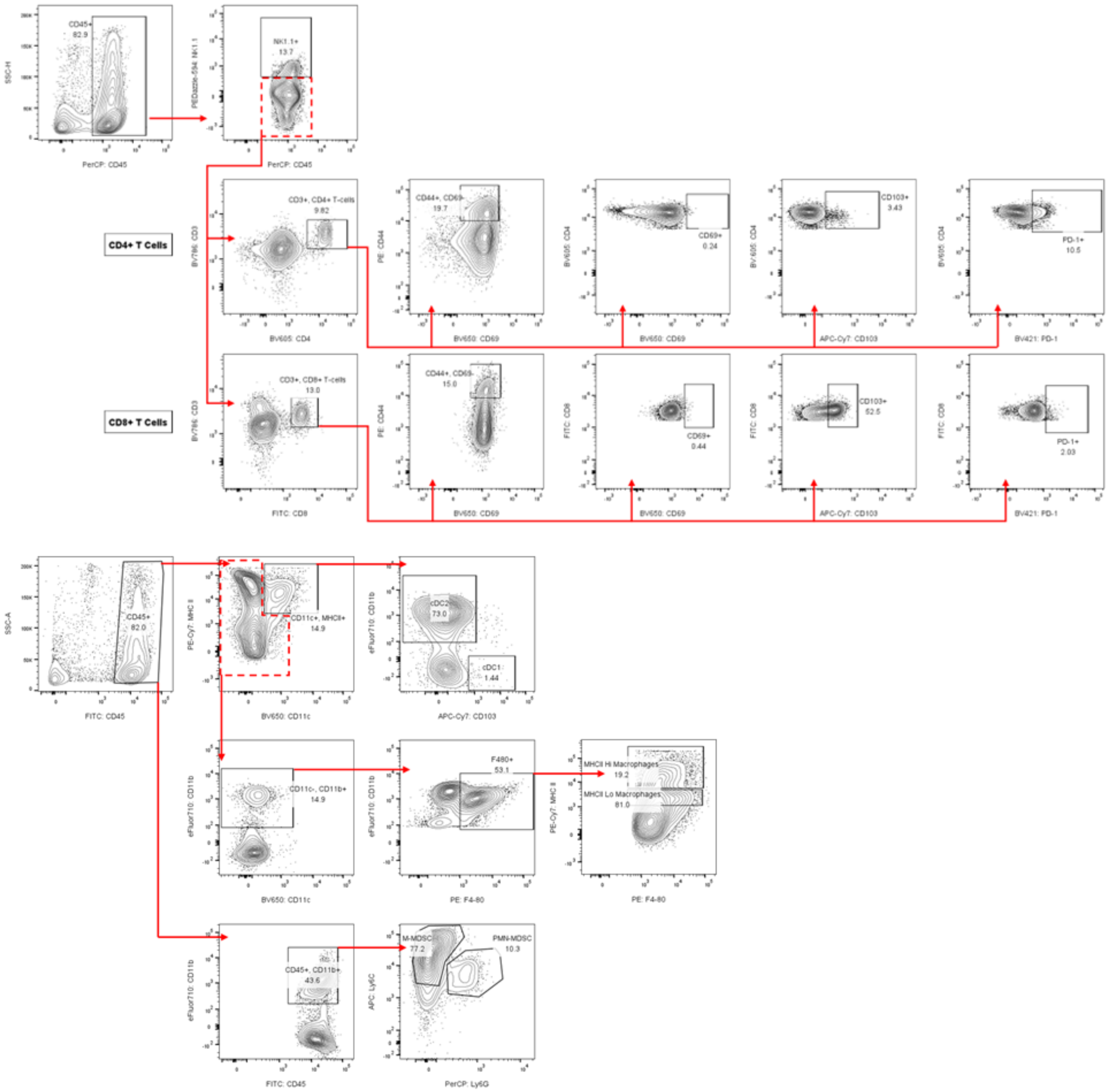

Supplemental Figure 1. 4T1 systemic immune profiling flow cytometry gating scheme.

Supplemental Figure 2.

E0771 Tumor Immune Profiling Flow Cytometry Gating Scheme

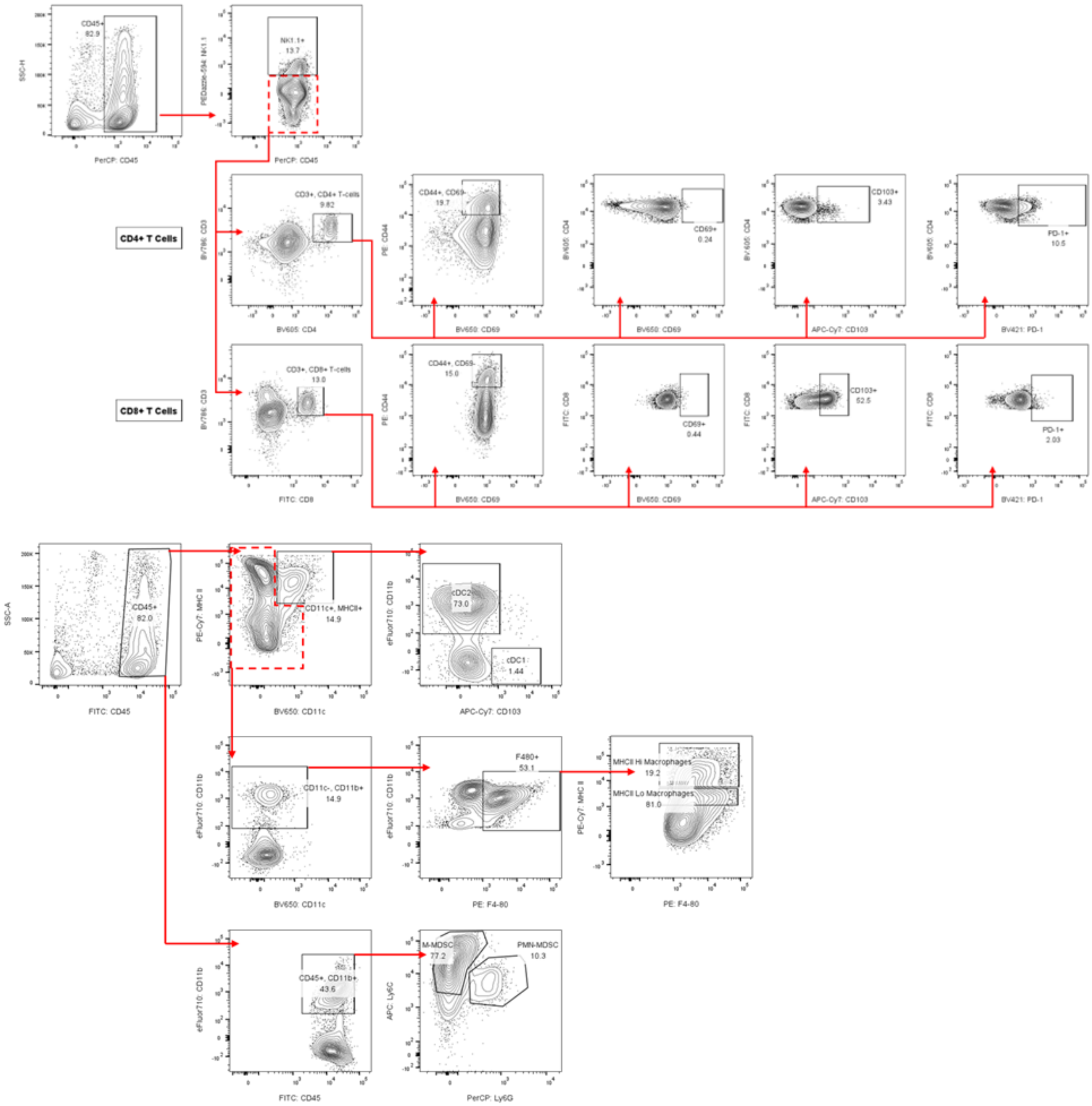

Supplemental Figure 2. E0771 tumor immune profiling flow cytometry gating scheme.

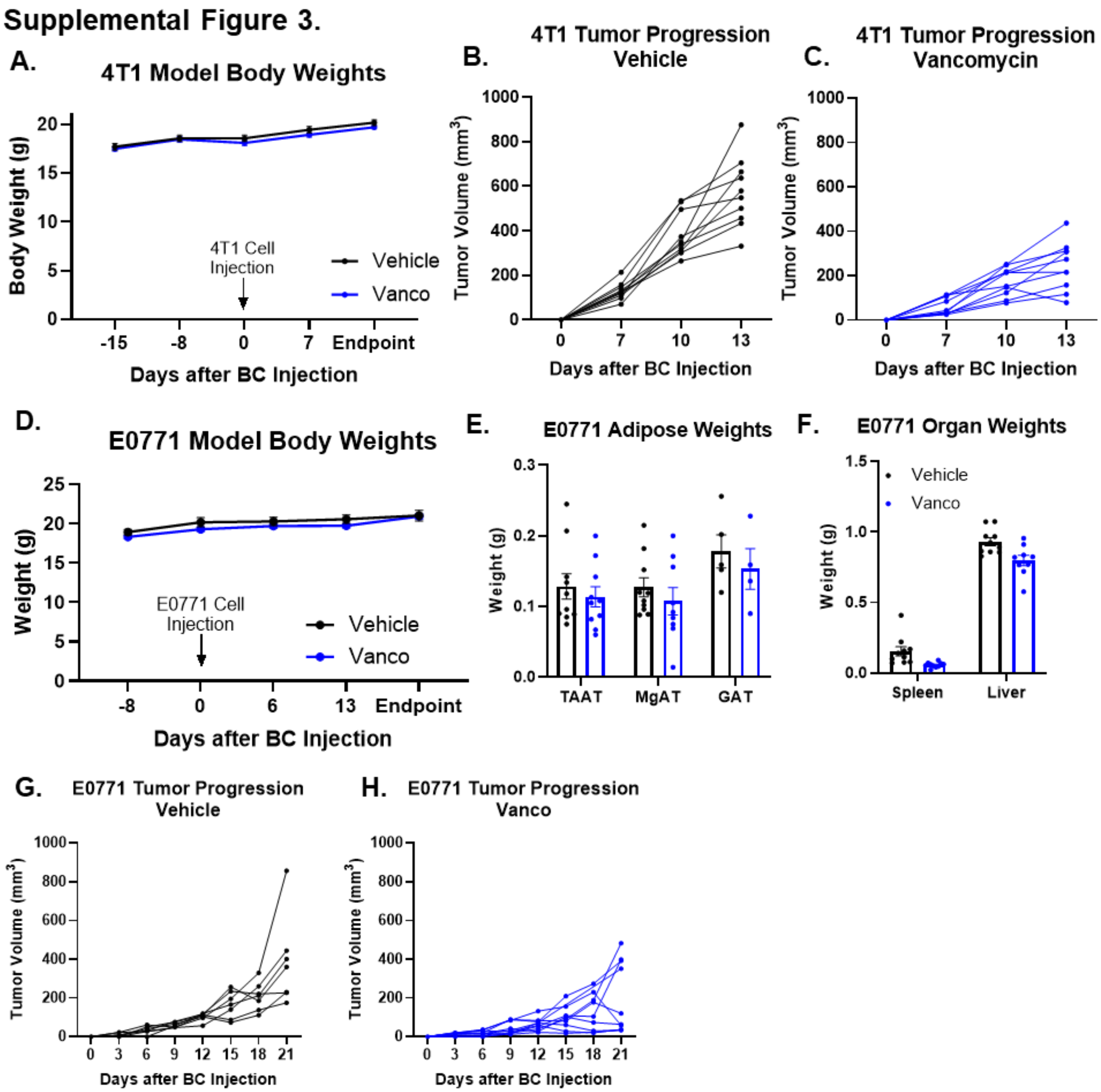

Supplemental Figure 3. Vancomycin did not impact mouse body weight or adiposity. (**A**) Body weights of BALB/cJ mice injected with 4T1 tumors were measured for duration of study with data presented as mean ± SEM with multi-effects analysis and Fisher's LSD correction. N = 10 per group. (**B-C**) 4T1 tumor progression graphed for individual mice in vehicle (**B**) and vancomycin (**C**) groups. (**D**) Body weights of C57BL/6J mice injected with E0771 tumors were measured for duration of study with data presented as mean ± SEM with multi-effects analysis and Fisher's LSD correction. N = 7-10 per group. (**E**) Excised adipose depot mass from E0771 model was determined at endpoint for tumor-associated adipose tissue (TAAT), mammary gland adipose tissue (MgAT), and gonadal adipose tissue (GAT). Data presented as mean ± SEM with two-way ANOVA. N = 7-10 per group. (**F**) Excised mass of spleens and livers from E0771 model was determined at endpoint. Data presented as mean ± SEM with two-way ANOVA. (**G-H**) E0771 tumor progression graphed for individual mice in vehicle (**G**) and vancomycin (**H**) groups.

Supplemental Figure 4.

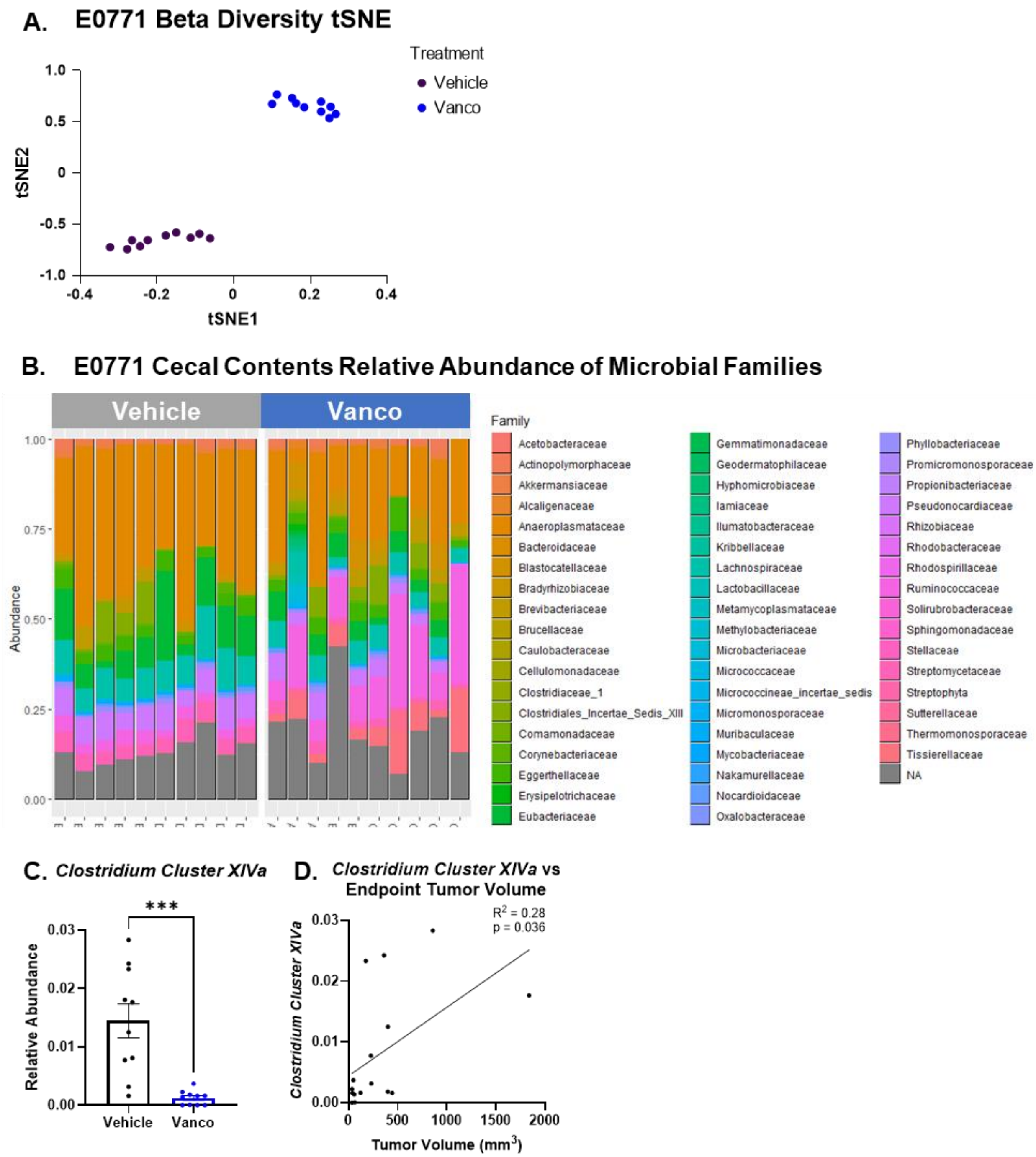

Supplemental Figure 4. Vancomycin reduced diversity in the gut microbiota of C57BL/6J mice injected with E0771 tumors. (**A**) Microbial beta diversity of cecal contents collected from E0771 model at endpoint is shown by principal component analysis of 16S microbiome sequencing with clustering determined by Bray-Curtis distances. N = 10 per group. (**B**) Relative abundance of microbial families is reported in cecal contents harvested at study endpoint. (**C**) Relative abundance of *Clostridium Cluster XIVa* in cecal contents is reported. Data presented as mean ± SEM with Student's *t* test for N = 10 per group. (**D**) Relative abundance of *Clostridium Cluster XIVa* positively correlated with endpoint E0771 tumor volume by MaAsLin2 analysis, $R^2 = 0.28$, $p = 0.036$, N = 20. ***$p < 0.001$.

Supplemental Figure 5.

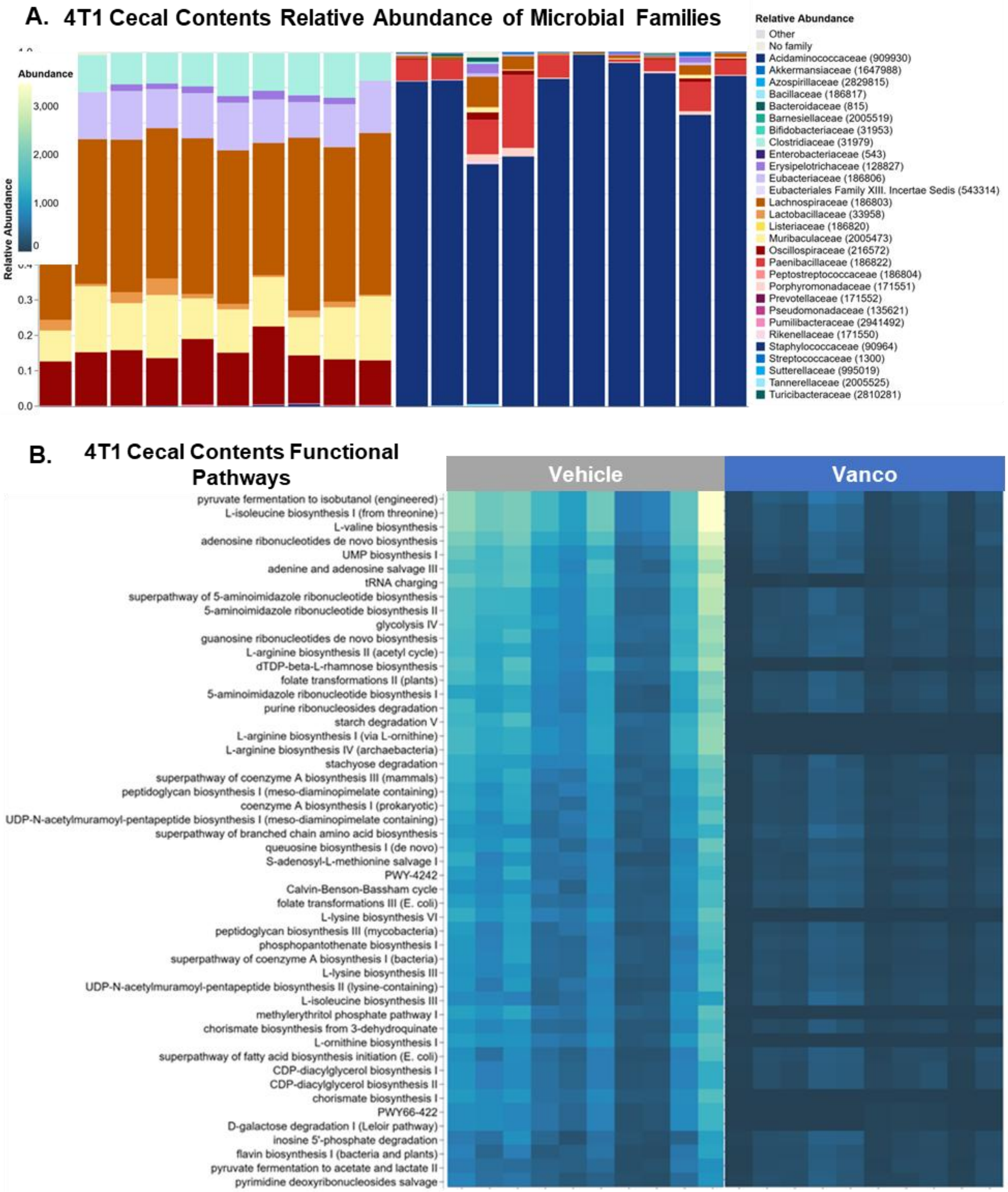

Supplemental Figure 5. Vancomycin downregulated expression of microbial functional pathways. (**A**) Relative abundance of microbial families is reported in 4T1 model cecal contents harvested at study endpoint. N = 10 per group. (**B**) The top 50 functional pathways significantly impacted by vancomycin treatment were identified by the MetaCyc database using the Humann3 pipeline and presented as row-mean-centered heatmap. N = 10 per group.
